# A Supramolecular Interaction of a Ruthenium Complex With Calf-Thymus DNA: A Ligand Binding Approach by NMR Spectroscopy

**DOI:** 10.3389/fchem.2019.00762

**Published:** 2019-11-08

**Authors:** Flávio Vinícius Crizóstomo Kock, Analu Rocha Costa, Katia Mara de Oliveira, Alzir Azevedo Batista, Antônio Gilberto Ferreira, Tiago Venâncio

**Affiliations:** ^1^Laboratory of Nuclear Magnetic Resonance, Department of Chemistry, Federal University of São Carlos, São Carlos, Brazil; ^2^Laboratory of Structure and Reactivity of Inorganic Compounds, Department of Chemistry, Federal University of São Carlos, São Carlos, Brazil

**Keywords:** binding interactions, lawsone, ruthenium complex, anti-cancer, CT-DNA, NMR

## Abstract

Lawsone itself exhibits interesting biological activities, and its complexation with a metal center can improve the potency. In this context a cytotoxic Ru-complex, [Ru(law)(dppb)(bipy)] (law = lawsone, dppb = 1,4-bis(diphenylphosphino)butane and bipy = 2,2′-bipyridine), named as CBLAU, was prepared as reported. In this work, NMR binding-target studies were performed to bring to light the most accessible interaction sites of this Ru-complex toward Calf-Thymus DNA (CT-DNA, used as a model), in a similar approach used for other metallic complexes with anti-cancer activity, such as cisplatin and carboplatin. Advanced and robust NMR binding-target studies, among them Saturation Transfer Difference (STD)-NMR and longitudinal relaxometry (T_1_), were explored. The ^1^H and ^31^P -NMR data indicate that the structure of Ru-complex remains preserved in the presence of CT-DNA, and some linewidth broadening is also observed for all the signals, pointing out some interaction. Looking at the binding efficiency, the T_1_ values are highly influenced by the formation of the CBLAU-DNA adduct, decreasing from 11.4 s (without DNA) to 1.4 s (with DNA), where the difference is bigger for the lawsone protons. Besides, the STD-NMR titration experiments revealed a stronger interaction (K_D_ = 5.9 mM) for CBLAU-DNA in comparison to non-complexed lawsone-DNA (K_D_ = 34.0 mM). The epitope map, obtained by STD-NMR, shows that aromatic protons from the complexed lawsone exhibits higher saturation transfer, in comparison to other Ru-ligands (DPPB and bipy), suggesting the supramolecular contact with CT-DNA takes place by the lawsone face of the Ru-complex, possibly by a spatial π-π stacking involving π-bonds on nucleic acids segments of the DNA chain and the naphthoquinone group.

## Introduction

Cancer is among the major public health problems worldwide and is responsible for millions of deaths, only in the United States (Siegel et al., 2019). Many biological macromolecular targets are involved in the complex mechanism of the cancer development, and nucleic acids, such as DNA, are one of these targets. The action of traditional chemotherapeutics based on metallic complexes (metallodrugs), for example cisplatin and carboplatin, involves their interaction with the DNA. Hence, the search for new metallic complexes with anti-tumor activity is one of the most important research field in bioinorganic chemistry. Regarding this action mechanism, studies aiming to deep the knowledge about the intermolecular interaction between new potential drugs and biological targets are relevant, because it leads the design of new potent and selective anti-cancer candidates.

A strategy used to design new chemotherapeutics candidates and improve its anti-tumoral activity, consists on the complexation of some metallic centers, among them, silver (I) (Ali et al., 2013; Engelbrecht et al., 2018; Hussaini et al., 2019), gold (I)/(III) (Yeo et al., 2018; Dabiri et al., 2019; Hussaini et al., 2019), copper (II) (Khan et al., 2017; Zhang et al., 2018; Chen et al., 2019), and platinum (II) (Hua et al., 2019; Hussaini et al., 2019; Makovec, 2019) with a wide-range of ligands and also with natural molecules that already demonstrate some anti-cancer features, such as quinoline, flavones, and naphthoquinones (Kosmider and Osiecka, 2004; Lu et al., 2016; Oliveira et al., 2017a; Wang et al., 2018; Grandis et al., 2019). In this scenario, ruthenium takes special interests due to the possibility to reach several different metallic-arrangements, supplying distinct reactivities and applications (Oliveira et al., 2017b; Hussaini et al., 2019; Roy et al., 2019).

In this work, the ligand (2-hydroxy-1,4-naphthoquinone), a naphthoquinone with anti-viral, anti-fungal, anti-parasitic, anti-microbial, and anti-cancer biological features well-described in literature (Oliveira et al., 2017a) and the Ru/lawsone complex, [Ru(law)(dppb)(bipy)] (law = lawsone, dppb = 1,4-bis(diphenylphosphino)butane and bipy = 2,2′-bipyridine), named as CBLAU were fully investigated by NMR spectroscopy. The action of this potential metallodrug against tumor cell lines, among them, DU-145 (prostate cancer cells), MCF-7 (breast cancer cells), A549 (lung cancer cells) founds reported in the literature as well as its potential to induce the tumor cells apoptosis (Oliveira et al., 2017a). Considering that for all these cases, DNA is a possible and relevant biological target, similarly that occurs for cisplatin and/or carboplatin that acts binding covalently with pairs of nitrogenous DNA bases (Oliveira et al., 2017a), the intermolecular interaction of this new candidate (CBLAU) toward this biological target, is investigated in this research. In this work the intermolecular binding interactions will be investigated by NMR approaches, aiming to probe the intermolecular contact in the atomic level. These efforts can be reached using NMR techniques able to provide a reliable answer at molecular level (Viegas et al., 2011; Tanoli et al., 2015, 2018; Monaco et al., 2018).

Among several other techniques used for probing binding-targets interaction, among them circular dichroism, viscosimetry, and fluorescence (Oliveira et al., 2017a,b, 2019; Villareal et al., 2017; Gagini et al., 2018; Grandis et al., 2019), NMR-based methods is a unique spectroscopy able to supply in atomic level reliable answers for binding affinities studies in aqueous media (Tanoli et al., 2015, 2018). In addition, this experimental approach requires a very low concentration of protein without isotopic labeling, with any prior knowledge of its structure and/or function and does not have restriction on the protein size (Mayer and Meyer, 1999, 2001; Angulo et al., 2010; Angulo and Nieto, 2011; Viegas et al., 2011; Monaco et al., 2018). Based on the aforementioned features, these NMR experiments are the most routinely used techniques for drug/biological target recognition processes, through the knowledge about the spatial interaction, binding epitope mapping (GEM), and determination of dissociation constants (K_D_), supplying an unequivocal grasp about the intermolecular interactions, where most of the others analytical methods are unable to provide satisfactory answers (Mayer and Meyer, 1999, 2001; Angulo et al., 2010; Angulo and Nieto, 2011; Viegas et al., 2011; Monaco et al., 2018; Nepravishta et al., 2019).

Therefore, herein, we use NMR binding-target interaction approaches, among them ^1^H- and ^31^P-NMR, relaxometric experiments, Saturation Transfer Difference (STD)-NMR for scrutiny at atomic level the intermolecular interaction between the potential anti-cancer candidate (CBLAU) toward DNA. To the best of our knowledge few papers devote special attention on a detailed application of NMR spectroscopy to characterize the interaction of metallic complexes with macromolecular targets. As a result, we expect to introduce detailed NMR studies in order to open a new possibility of frontiers researches in bioinorganic chemistry, aiming to rationalize the designing of new metallodrugs based chemotherapeutics, with improved potency and selectivity against cancer, which still remains a challenge.

## Experimental

### Sample Preparation

Calf-thymus DNA (CT-DNA) was purchased from Sigma-Aldrich Ltd (Brazil) and used without previous purification. A 1.078 mM solution of CT-DNA was prepared by dissolving an adequate weight of this macromolecule in a deuterated phosphate buffer. The pH 7.2 phosphate D_2_O buffer was prepared by dissolving disodium hydrogen phosphate (Na_2_HPO_4_.12H_2_O, 0.1180 g) and sodium dihydrogen phosphate (NaH_2_PO_4_.2H_2_O, 0.0217 g) in 10.00 mL D_2_O (99.9%, Cambridge Isotopes Laboratories, Inc. with pH 7.2) to make a 0.046 M solution. The (CBLAU)(${\text{BF}}_{4}^{-}$) complex was dissolved in a 5:95% v/v of DMSO (99.8% Cambridge Isotopes Laboratories, Inc.) and deuterated buffer respectively. ${\text{BF}}_{4}^{-}$ was used as a counter ion together with 5% of DMSO-d6 to increase the solubility of the Ru-complex in aqueous solution. All ^1^H NMR and STD-NMR spectra were recorded using a 1:100 (DNA:CBLAU) molar ratio, in a final volume of 500 μL transferred to a 5 mm NMR tube (Norrel, Inc. USA). For the epitope mapping experiment, the final concentration of CBLAU was 5 mM and the concentration for the CT-DNA was 50 μM. On the other hand, for the concentration-dependent STD-NMR experiment, the CBLAU and lawsone concentrations were ranged from 5 to 1 mM. In these experiments, the CT-DNA concentration was fixed in 50 μM. For these studies, 7 independent STD-NMR experiments were acquired for each concentration.

### NMR Spectroscopy

All ^1^H-NMR based experiments were recorded on a Bruker Avance III 14.1 T spectrometer equipped with a TCI cryoprobe and the ^31^P-NMR experiments were performed on a Bruker Avance III 9.4 T spectrometer. The proton resonance frequencies for both magnetic fields are 600 and 400 MHz, respectively. Data acquisition and processing were performed with the Bruker software Topspin 3.5 pl. 7 version installed in the spectrometer. All the experiments were done at 298 K.

### One-Dimensional NMR (^1^H and ^31^P Experiments)

The ^1^H-spectra were acquired using a standard Bruker presaturation pulse sequence (zgesgp) pulses length of 9.57 us and 19.14 us for the 90° and 180°, respectively, with a gradient length pulse of 2,000 us. Besides, other experimental conditions were acquisition time of 4.63 s (32 K points), recycle delay of 4 s, spectral window of 20.02 ppm, and accumulation of 128 transients. For the acquisition of ^31^P-spectra a standard BRUKER pulse sequence (zgpg30) was used with a pulse length of 25.0 us and 50 us for the 90° and 180°, respectively. In addition, a spectral width of 289.40 ppm, recycle delay of 0.1 s (32 K points) were used and a total of 131,072 transients were co-added.

### Relaxometric Experiments

The relaxometric experiments were acquired using the standard Bruker pulse sequence inversion-recovery (t1ir), spectral window of 15.01 ppm, acquisition time of 1.8 s (32 K points), pulses lengths of 9.35 us and 18.70 us for the 90° and 180°, 16 transients and exponentially ranging the time between the pulses (τ) from 0.001 to 30 s. For the longitudinal relaxation time (T_1_) constant determination, was used the monoexponential fitting for the experimental obtained data, using for this purpose the software Topspin 3.5 pl.7.

### STD-NMR

These experiments were recorded by using a Bruker standard pulse sequence with water supression (stddiffesgp.3), with a pseudo-2D setup through an intervaled acquisition of an *on-* and *off-*resonance experiment. The water suppression was done under excitation sculpting scheme, properly optimized. Selective irradiation (during saturation time) of CT-DNA was achieved using a train of soft Gaussian-shaped pulses, with attenuation level of 45–55 dB, 50 ms of length and separated by a 2 ms interval. The frequency chosen for an adequate selective irradiation of CT-DNA was 2289.266 Hz (3.8 ppm) for *on-*resonance and 30,000 Hz (50 ppm) for *off-*resonance. The STD build up curve was acquired by ranging the saturation time between 0.5 and 10 s, and recording 14 independent experiments. Finally, the STD amplification factor (A_STD_) was calculated by accounting the signal intensity of the STD difference spectrum relative to the reference (off-resonance) according to Equation (1; Mayer and Meyer, 1999).

(1) $$\begin{array}{r} A_{STD}=\frac{I_{0}-I_{STD}}{I_{0}}\;\times \;\frac{\left[L\right]}{\left[P\right]} \end{array}$$

For the determination of the relative percentage of A_STD_, the proton signal with biggest integral area received the value of 100% and the other signals were normalized with respect to this signal. For the dissociation constant (K_D_) estimation, seven STD-NMR concentration-based experiments were performed. The results were plotted using the Equation 2, and the reciprocal of this equation (Lineweaver-Burk plot) was employed for estimation of K_D_.

(2) $$\begin{array}{r} A_{STD}=\frac{\alpha_{STD}\left[L\right]}{K_{D}+\left[L\right]} \end{array}$$

In this equation, A_STD_ corresponds to STD signal intensity, α_*STD*_ is the maximum STD intensity assuming infinite concentration, and [L] is the ligand concentration. After getting the A_STD_ and α_*STD*_ values, the curves A_STD_ vs. concentration were built up, linearized and fitted using a linear fit employing the MS Excel (2007) and plotted using the software Origin, version 8.0.

## Results and Discussions

### Signal Assignment

**Figure 1:**
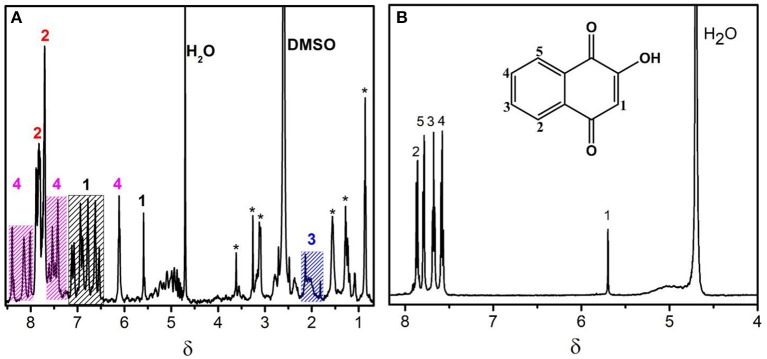
^1^H-NMR spectra obtained at 600 MHz (for ^1^H nucleus) and at 298 K for the characterization of the Ru-complex CBLAU **(A)** and the free ligand (lawsone) in **(B)**.

The ^1^H NMR signal assignment for CBLAU and lawsone are shown in Figures 1A,B, respectively. Due to the aromatic character of the ligands, the NMR region above 6.5 ppm at Figure 1A exhibits a series of superimposed signals. Despite this spectral feature, the signal discrimination can be done regarding each individual ligand. The set of signals numbered as 1 (black), from 6.8 to 7.4 ppm and at 5.7 ppm, correspond to aromatic hydrogens from lawsone ligand. Moreover, the intense signals assigned by the number 2 (red), from 7.6 to 7.9 ppm are attributed to hydrogens present on DPPB ligand. The signals numbered by 3 (blue), close to 2.0 ppm correspond to aliphatic hydrogens (butyl unit) present on DPPB ligand. To conclude, the set of signals numbered as 4 (pink), above 8.0 ppm and from 7.5 to 7.8 ppm, correspond to hydrogens from Bipy ligand. The presence of some impurities can be observed by the signals assigned with asterisks. These impurities remain even after the laborious process of CBLAU purification. Despite the presence of these residues, they do not interfere in the interaction between CBLAU and CT-DNA, as it will be shown in the following discussions. The Figure 1B shows the ^1^H-NMR spectrum for the lawsone ligand. On this spectrum, it is clearly possible to observe 5 signals. The hydrogens 2–5 correspond to the aromatic from 7.5 to 7.8 ppm, as indicated. The signal at 5.7 ppm corresponds to vinylic proton, assigned by the number 1.

**Figure 2:**
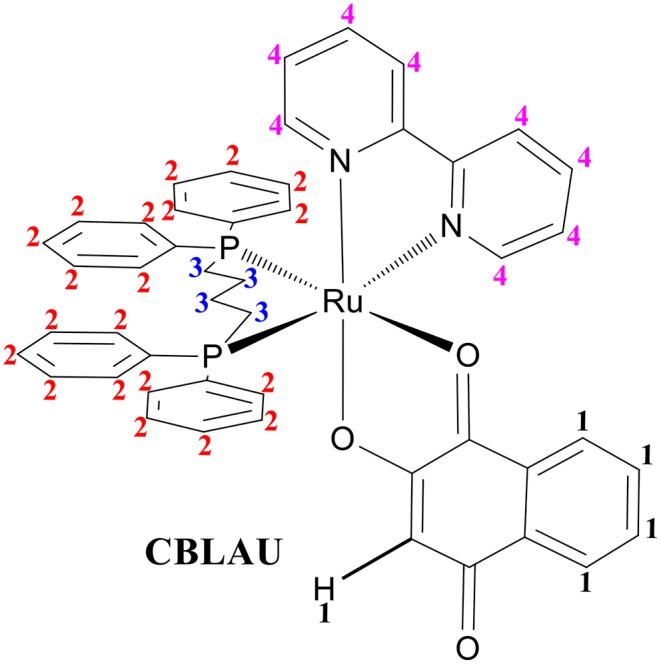
Chemical structure for the Ru-complex (CBLAU) addressed in this work: the numbers 1 (black) correspond to the ligand lawsone; the number 2 (red) to aromatic protons of DPPB ligand; the number 3 (blue) to aliphatic hydrogens (butyl unit) between the two DPPB units and the number 4 (pink) to the hydrogens from the Bipy ligand.

On Figure 2 the ^1^H NMR signal assignment can be better visualized on the chemical structure for CBLAU, highlighting the chemical groups responsible for each signal observed on the NMR spectra previously presented in the Figure 1A. The set of these results (Figures 1, 2) will be helpful on the subsequent binding target elucidation studies, providing an atomic comprehension about the contribution of each ligand group present on CBLAU and lawsone responsible by the efficient intermolecular interaction toward the CT-DNA strand.

### Interaction With Calf Thymus DNA

**Figure 3:**
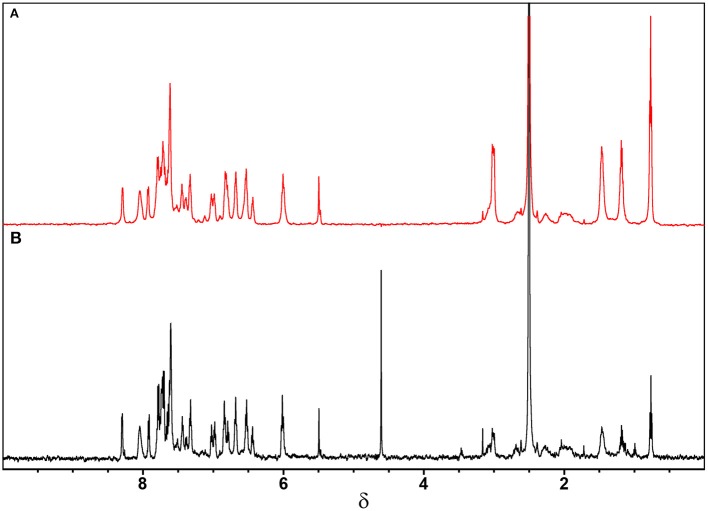
^1^H**-**NMR spectra obtained for CBLAU (5 mM): **(A)** with (red) and **(B)** without (black) CT-DNA (50 μM) at 600 MHz (for ^1^H nucleus) and 298 K.

In the first approach (Figure 3), the ^1^H-NMR spectra were obtained for the Ru-complex CBLAU in the presence of CT-DNA (red) and without CT-DNA (black) aiming to reach a preliminary picture about the interactions, based on some chemical shift variation and/or linewidth broadening. Interestingly, it is not observed any significant change in the NMR spectrum profile due to the presence of DNA. Some linewidth broadening is observed on the spectrum without CT-DNA (black) can be attributed to the complex size, its low solubility and the presence of some quadrupolar component present on some active NMR Ru-isotopes (Levitt, 2008; Keller, 2011). In the presence of CT-DNA, any change can be observed.

**Figure 4:**
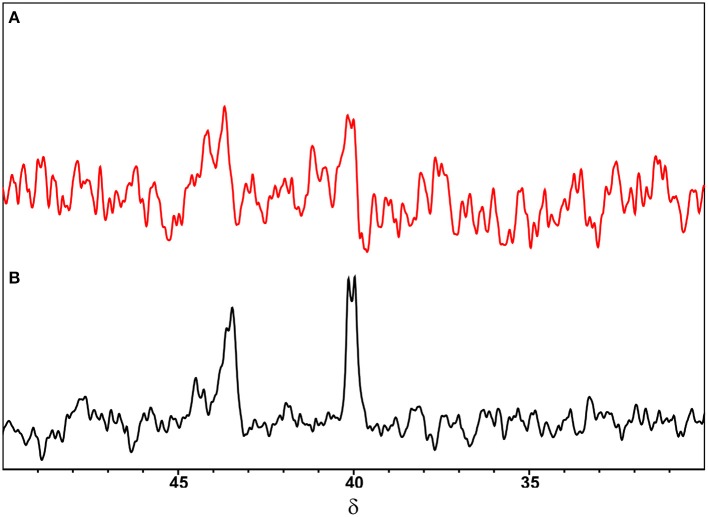
^31^P-NMR spectra obtained for CBLAU (5 mM): **(A)** with (red) and **(B)** without (black) CT-DNA (50 μM) at 161.96 MHz (for ^31^P nucleus) in DMSO:buffer (5:95 v/v) at 298 K.

Regarding these ^1^H-NMR results, the intermolecular interaction between CBLAU toward CT-DNA was investigated by ^31^P-NMR, aiming to scrutiny the influence of presence of CT-DNA on the DPPB ligand, and the preservation of the Ru-complex. For this purpose, two ^31^P-NMR spectra for CBLAU were collected, with and without CT-DNA. The resulting spectra are shown on the Figure 4. In the black line it is can be observed the ^31^P-NMR signal obtained for the CBLAU at 298 K in DMSO:buffer (5:95 v/v), without CT-DNA. The spectrum in red represents the ^31^P-NMR signal obtained for CBLAU in the presence of CT-DNA. By comparing the spectra, it is clearly observed that the presence of the macromolecule is responsible for the increasing of the ^31^P linewidth. Besides, the signal intensity is reduced, leading to confirm that the interaction between CBLAU and CT-DNA is highly important and demonstrate that for this case, ^31^P-NMR is more sensitive to investigate this binding interaction in comparison to ^1^H-NMR.

Therefore, from the analysis of ^1^H-NMR spectra it is possible to suggest that the interaction of CBLAU with CT-DNA is weak and possibly takes place through a spatial van-der-Waals intermolecular force. In fact, this type analysis is very preliminary and not conclusive, since the chemical shift variation, as well as the linewidth broadening, can be related to several other effects, such as pH and temperature variation. To circumvent this limitation other robust NMR methods can be better explored, such as NMR-relaxometry and STD-NMR for discriminate at atomic level the binding target intermolecular interactions. These NMR approaches will be explored in the later discussions.

The relaxometric, and particularly the longitudinal relaxation time, is highly useful as a preliminary estimative about the binding-target efficiency, since the longitudinal relaxation times vary according to changes in the molecular mobility. For this purpose, ^1^H T_1_ relaxation times were determined for the Ru-complex CBLAU with and without CT-DNA. The experimental data showed that the average longitudinal relaxation (T_1_) obtained for CBLAU (11.41 s) is highly influenced, due to the final CBLAU/CT-DNA adduct formation (1.40 s) in solution. These variations can be attributed to the reduction on the molecular mobility associated to CBLAU under the presence of CT-DNA, which is an extremely large macromolecule (8.41 × 10^6^ g.mol^−1^) that increases the viscosity of the solution (Porsch et al., 2009). Therefore, these results point out that CBLAU interacts with CT-DNA. The detailed results are listed on Table 1, for all the groups complexed with metallic center. The complexed lawsone and bipyridine are mostly affected by the presence of CT-DNA and these findings indicate that these groups exhibit a shorter contact with CT-DNA surface. The formation of the supramolecular adducts, CBLAU/CT-DNA obeys a binding equilibrium, where the lawsone and bipy face of CBLAU seems to be more involved in the intermolecular contact.

**Table 1:** ^1^H T_1_ relaxation times determined for the Ru-complex CBLAU in solution (5 mM) and in the presence of CT-DNA (50 μM). Results collected at 298 K (600 MHz).

**Group**	**T_**1**_ (s) with CT-DNA**	**T_**1**_ (s) without CT-DNA**	**ΔT_**1**_ (s)**
Lawsone	1.4	11.5	10.1
Bipy	1.1	11.4	10.3
DPPB	1.4	11.1	9.7
Butane branch	1.8	11.6	9.8

**Figure 5:**
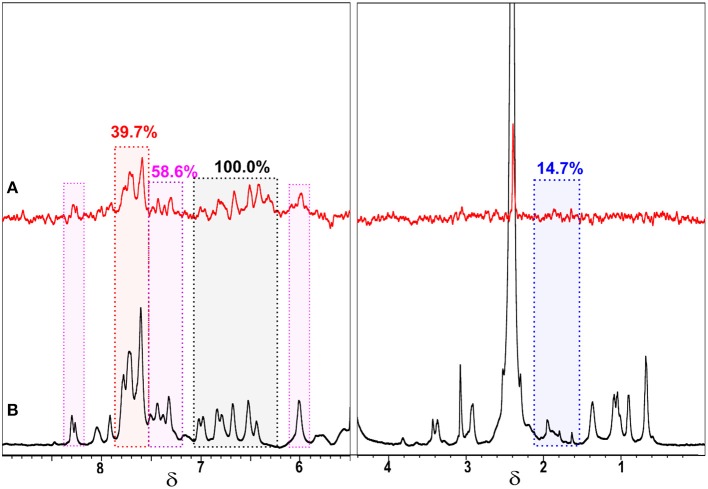
Off-resonance STD spectrum **(A)** and STD diff-spectrum **(B)** obtained by applying 10.0 s of saturation time and used for the calculation of A_STD_ and consequent epitope map, highlighting the contribution (proximity) of each group present on CBLAU (5 mM), bipy (58.6%) (pink), butyl protons between the DPPB units (14.7%) (blue), lawsone (100.0%) (black), and DPPB (39.7%) (red) to CT-DNA (50 μM) surface.

Despite these positive results of these relaxometry based binding-target experiments in demonstrating the interaction of CBLAU with CT-DNA, the T_1_ relaxation times could change due to other effects, such as the viscosity variation. On the other hand, the T_1_ variation is different for each group complexed to ruthenium, as mentioned. In this scenario, the interaction between CBLAU and CT-DNA was investigated by STD-NMR experiments (Figure 5), which is more robust, and depends exclusively on the spatial contact of the molecules involved in the interaction. This experiment allows to estimate not only the most accessible sites of binding contact present but supplies the intensity of these interactions. The epitope map, which describes the spatial proximities between the small molecule (CBLAU and lawsone) and the macromolecular target (CT-DNA), was built up from the acquisition of 14 STD-NMR experiments, ranging the saturation times from 0 to 10 s. Once the data was collected, the STD amplification factor (A_STD_) was calculated using the STD_off−resonance_ (Figure 5A) and STD_diff_ (Figure 5B) spectra, and performing the ratio of these spectra, in agreement to Equation (1). For the signal with higher A_STD_ value was attributed the percentage of 100% and for the other protons, the A_STD_ values were calculated with relation to this initial consideration. Besides, the signal intensity, as observed in these figures, does not only reflects the relative amount of saturation transferred from CT-DNA to CBLAU *via* cross-relaxation (spin-diffusion), but also reflects the relative proximity of a respective proton from CBLAU to the biological target surface on the final adduct (Mayer and Meyer, 1999, 2001; Angulo et al., 2010; Angulo and Nieto, 2011; Viegas et al., 2011; Monaco et al., 2018). In addition, it is important to stress that, the signal intensities in STD-NMR spectrum strongly depends on the equilibrium established between the species involved in the interaction, with similarities to the enzymatic inhibition equilibrium. Therefore, as in agreement to literature, two factors are important in these terms, one is the concentration of the small molecule and the second is the saturation transfer from the macromolecule to the small molecule (Viegas et al., 2011; Tanoli et al., 2015, 2018; Monaco et al., 2018). In this context, it is expected that longer saturation times improve the saturation transfer in the limit governed by the timescale of the contact between the protons from CBLAU closer to CT-DNA surface. Thus, as the CBLAU concentrations increase, the CT-DNA surface reaches some saturation and, as response, the STD signal intensities should increase (Tanoli et al., 2015, 2018).

**Figure 6:**
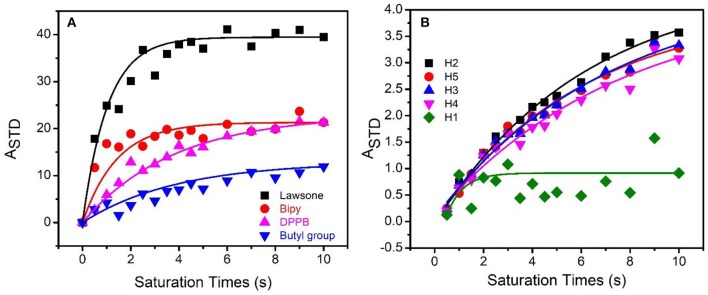
Built-up curves of A_STD_ against saturation time at 298 K and 600 MHz (^1^H nucleus) for CBLAU (5 mM) **(A**) and for the individual lawsone (5 mM) **(B)** in the presence of CT-DNA (50 μM).

Regarding the dependence of the equilibrium with the timescale of the intermolecular contact between CBLAU and CT-DNA several STD experiments were collected by varying the saturation time. Therefore, analyzing the STD_off−resonance_ and STD_diff_ spectra a built-up curve was obtained ranging the saturation time from 0 to 10.0 s for each CBLAU group, as shown in Figure 6A, where the exponential growth and the intensity of the curve plateau reflects the proximity of each group to the CT-DNA surface. This analysis allows to reach a map of the proximities between the Ru-complex and the CT-DNA surface, and this is known as epitope map. The results show that the lawsone group is the closest one (100.0%) in comparison to Bipy (58.6%), DPPB (39.7%), and butyl groups (between DPPB units), 14.7%. The epitope map shows that the interaction involves peripheral CBLAU protons, as expected, and an important finding is that the metallic complex structure is preserved, as previously observed by ^1^H-NMR data. But an initial question should be answered regarding the recognized biological activity of lawsone: does the complexation with a metallic center improve its activity? This is an important concerning to justify the Ru-complex preparation. At this point, several STD experiments were conducted for individual lawsone, following the same approach.

For the lawsone, STD experiments showed that the all aromatic protons are close to CT-DNA surface, as expected (Figure 6B). The proton 1 (green) refers to a lower (63.9%) A_STD_ value in comparison to the other protons, which A_STD_ values are quite similar: proton 2 (black) (100%), proton 3 (blue) (96.4%), proton 4 (pink) (94.3%), and proton 5 (red) (99.4%), respectively. Therefore, protons numbered from 2 to 5 are closer to CT-DNA and π-π stacking forces seem to govern the contact. Besides, these results reinforce that the presence of ligands containing aromatic groups on the design of anti-cancer candidates favors the contact with the DNA surface.

Interestingly, it is observed on Figure 6B that the A_STD_ values for the 2 to 5 protons of the non-Ru complexed lawsone exponentially increased until 10 s of saturation time, but differently to note for the CBLAU protons, the A_STD_ does not reach a stationary plateau, supposed at elevated saturation times conditions. These results indicate that besides these protons are closer to DNA, they are not completely saturated, in despite of the elevated saturation time, which is an additional indicative that the interaction of the non-Ru complexed lawsone with CT-DNA is weaker, in comparison to the Ru-complexed one. In addition, it is also observed for the proton 1 (green) on lawsone, that the A_STD_ values is more randomized in comparison to that one reached for another protons. These observations do not prevent the determination of A_STD_, but demonstrate that the resonances close to solvent signal (4.7 ppm) are more influenced by the solvent suppression scheme used, where the excitation profile associated to the pulse sequence used also reduce the solvent nearby signals.

**Figure 7:**
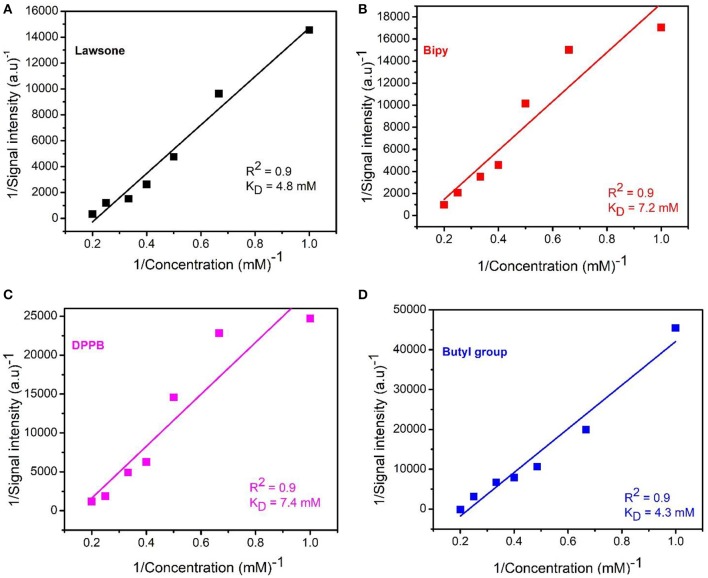
Lineweaver-Burk plot for the experimental STD-NMR concentration experiments obtained for CBLAU and used to estimate the dissociation constant (K_D_) for each group: lawsone (4.8 mM) **(A)**, bipy (7.2 mM) **(B)**, DPPB (7.4 mM) **(C)** and butyl group (4.3 mM) **(D)** and the average K_D_ value (5.9 mM) for this molecule (CBLAU) in interaction to CT-DNA.

Nevertheless, a second question remains unanswered: how strong is the interaction between CBLAU and CT-DNA? To explore the quantitative aspects of the equilibrium established with interaction, the dissociation constants (K_D_) were estimated for both cases: individual lawsone and Ru-complexed lawsone as in CBLAU. The estimation of K_D_ was performed by using several STD experiments, now varying the CBLAU concentration, in order to obtain a built-up curve, fitted by using a Lineweaver-Burk approach, as presented in the experimental section. Notably, the Ru-complexed lawsone exhibits an average dissociation constant of 5.9 mM (Figure 7), which is much smaller than the average K_D_ obtained for non-Ru complexed lawsone:CT-DNA supramolecular adduct (34.0 mM) (Figure S1). To conclude it is quite clear that when complexed, the lawsone exhibits a stronger interaction with CT-DNA. Therefore, these results put light on the previously reported biological results (Oliveira et al., 2017a; Grandis et al., 2019), demonstrating that the CBLAU complex interacts toward CT-DNA stronger than for non-Ru complexed lawsone.

## Conclusions

The interaction between a novel anti-cancer candidate and the biological target DNA was understood at atomic level by NMR-binding approaches. The relaxometric results demonstrated to be an efficient probe to monitor the formation of CBLAU/CT-DNA adduct in solution and the T1 variation is bigger for lawsone and bipyridine groups. Besides, it was also demonstrated that this binding target interaction have a weak nature (K_D_ = 5.9 mM), experimentally proving the pivotal role assumed by the spatial contact, such as hydrogen bonding, hydrophobic contacts, hydrophilic-hydrophobic interactions and π-π stacking on the final adduct arrangement. The studies involving only the non-complexed lawsone suggests that the binding mode toward DNA occur in a similar way, but the interaction intensity is notably lower (K_D_ = 34.0 mM) in comparison to the Ru-complexed one. These results also reinforce the search for new metallic complexes with organic molecules with recognized biological activity. The complexation with a metal can improves the interaction of these molecules with macromolecular biological targets. All these results put light on the reasons about the relevant biological features associated to this candidate against tumor cell lines DU-145 (prostate cancer cells), MCF-7 (breast cancer cells), A549 (lung cancer cells) as well as its potential to induce the tumor cells apoptosis. Therefore, we expect that these findings help the researchers working on bioinorganic to design new metallodrugs with improved activity and selectivity.

## Data Availability Statement

The datasets for this study are available on request to the corresponding author.

## Author Contributions

FK, AF, and TV designed the experiments. FK and TV generated and analyzed the experimental data. AC, KO, and AB designed and synthetized the complex CBLAU and provided the non-complexed lawsone. The manuscript was written by FK and TV. TV has supervised and finalized the final version of the manuscript.

### Conflict of Interest

The authors declare that the research was conducted in the absence of any commercial or financial relationships that could be construed as a potential conflict of interest.
