# Supplementary material for: A Supramolecular Interaction of a Ruthenium Complex With Calf-Thymus DNA: A Ligand Binding Approach by NMR Spectroscopy
Source: Front Chem. 2019 Nov 8;7:762. doi: 10.3389/fchem.2019.00762 (PMC6857657; doi:10.3389/fchem.2019.00762)
Supplement: Supplementary file 1 [file Table_1.DOCX]

A supramolecular interaction between ruthenium complex and Calf-Thymus DNA: a ligand binding approach by NMR spectroscopy

Flávio Vinícius Crizóstomo Kock^1^, Analu Rocha Costa^2^, Katia Mara de Oliveira^2^, Alzir Azevedo Batista^2^, Antônio Gilberto Ferreira^1^, Tiago Venâncio^1*^

^1^Laboratory of Nuclear Magnetic Resonance, Department of Chemistry, Federal University of São Carlos, São Carlos, Brazil.

^2^Laboratory of Structure and Reactivity of Inorganic Compounds, Department of Chemistry, Federal University of São Carlos, São Carlos, Brazil.

* Correspondence: venancio@ufscar.br

Supplementary Material

**Table of contents**

- Description of the synthesis and purification of the Ru-complex (CBLAU)
- Lineweaver-Burk plots for the titration STD-NMR experiments for non-complexed lawsone.

**Synthesis of [Ru(law)(dppb)(bipy)](PF_4_^-^)**

In a Schlenk flask 0.048 g (0.17 mol) of lawsone were dissolved in a mixture (1:1) of methanol and dichloromethane as solvent, and just after the triethylamine was added. Later, 0.10 g (0.13 mmol) of the *cis*-[RuCl_2_(dppb)(bipy)] precursor and 0.048 g (0.26 mmol) of tetrafluoroborate (BF_4_^-^) were added and the reaction mixture was kept overnight at room temperature (23 ^o^C). The volume was reduced to approximately 3 mL and the mixture was kept under stirring until the formation of a purple solid. The final solid product was then filtered and washed with distilled water and ethyl alcohol.


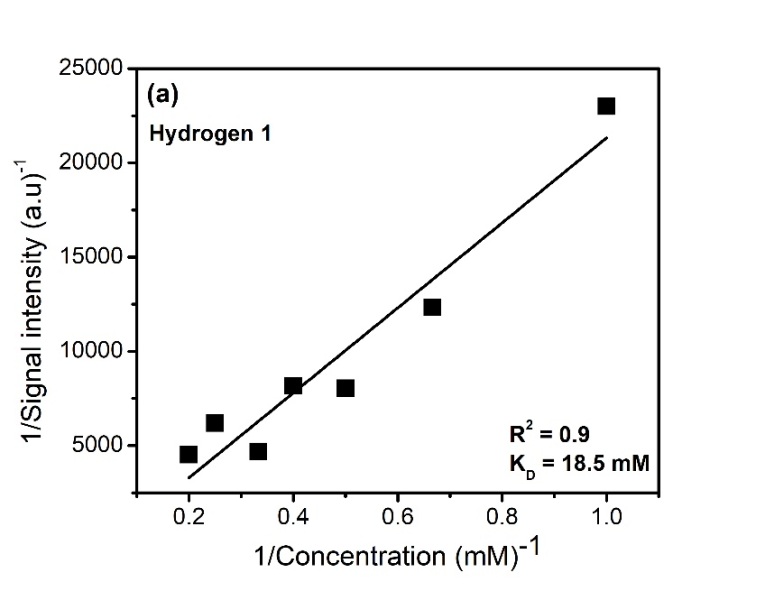

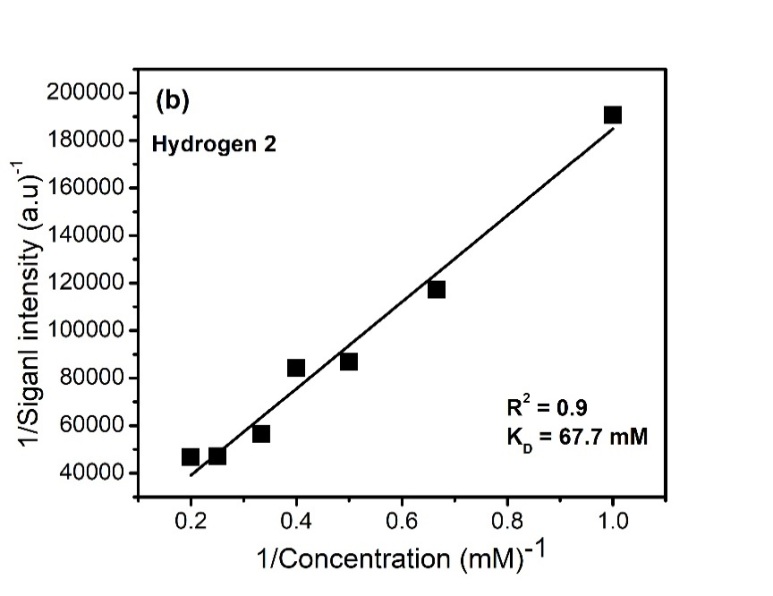


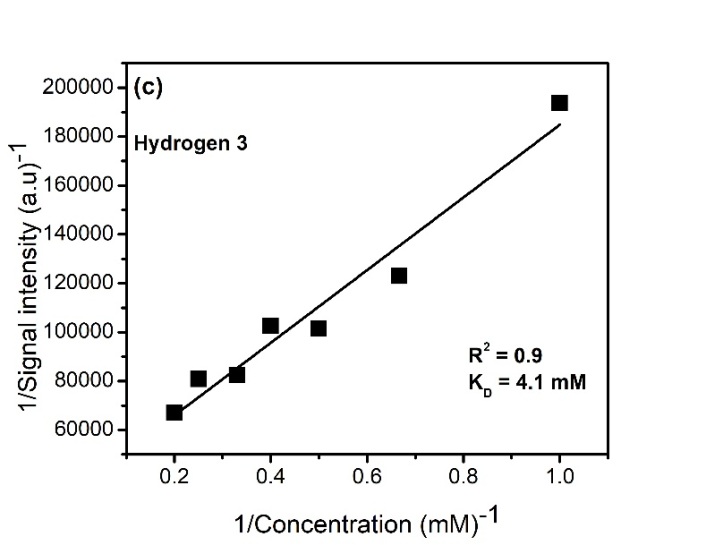

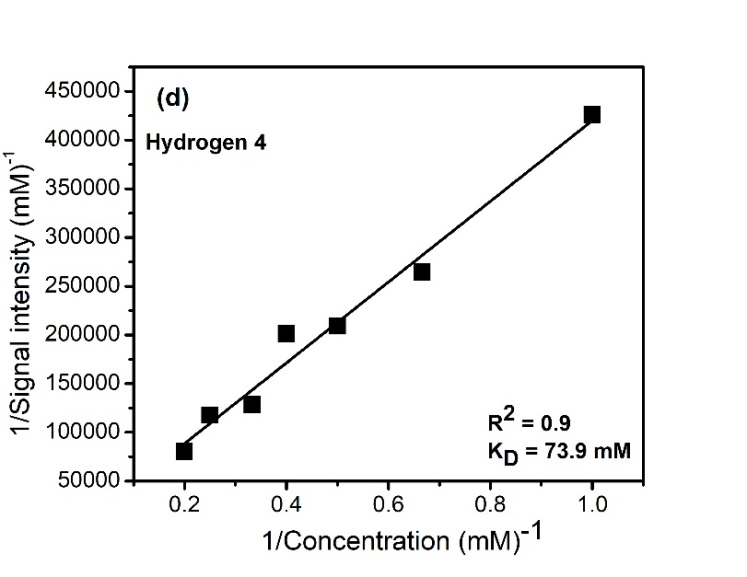


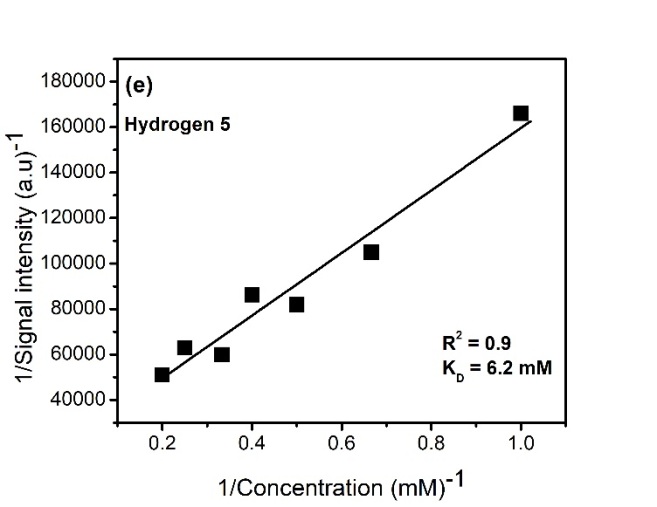


**Figure S1.** Lineweaver-Burk plots for the titration STD-NMR experiments obtained for the non-Ru complexed lawsone and used for the calculations of average dissociation constant value (K_D_ = 34.0 mM) for the interaction with CT-DNA.
